# Molecular Characterization of *Leptospira* Species among Patients with Acute Undifferentiated Febrile Illness from the Municipality of Villeta, Colombia

**DOI:** 10.3390/tropicalmed9080168

**Published:** 2024-07-25

**Authors:** Carlos Ramiro Silva-Ramos, J. Manuel Matiz-González, Juliana Gil-Mora, Heidy-C. Martínez Díaz, Álvaro A. Faccini-Martínez, Claudia Cuervo, Peter C. Melby, Patricia V. Aguilar, Miguel M. Cabada, Juan David Rodas, Marylin Hidalgo

**Affiliations:** 1 Grupo de Enfermedades Infecciosas, Departamento de Microbiología, Facultad de Ciencias, Pontificia Universidad Javeriana, Bogotá 110231, Colombia; juan.matiz@javeriana.edu.co (J.M.M.-G.); juliana.gil@javeriana.edu.co (J.G.-M.); h-martinez@javeriana.edu.co (H.-C.M.D.); claudia.cuervo@javeriana.edu.co (C.C.); 2 Molecular Genetics and Antimicrobial Resistance Unit, Universidad El Bosque, Bogotá 110121, Colombia; 3 Servicio de Infectología, Hospital Militar Central, Bogotá 110110, Colombia; afaccini@gmail.com; 4 Facultad de Medicina, Universidad Militar Nueva Granada, Bogotá 110111, Colombia; 5 Servicios y Asesorías en Infectología—SAI, Bogotá 110110, Colombia; 6 Division of Infectious Diseases, Department of Internal Medicine, University of Texas Medical Branch, Galveston, TX 77555, USA; pcmelby@utmb.edu (P.C.M.); micabada@utmb.edu (M.M.C.); 7 Center for Tropical Diseases, University of Texas Medical Branch, Galveston, TX 77555, USA; pvaguila@utmb.edu; 8 Department of Pathology, University of Texas Medical Branch, Galveston, TX 77555, USA; 9 Grupo de Investigación en Ciencias Veterinarias Centauro, Universidad de Antioquia, Medellín 050010, Colombia; jdavid.rodas@udea.edu.co

**Keywords:** *Leptospira*, leptospirosis, febrile patients, acute undifferentiated febrile illness, Colombia, molecular detection, phylogeny

## Abstract

*Leptospira* is a bacterial genus that includes several pathogenic species related to leptospirosis. In Colombia, leptospirosis is a mandatorily reported disease, widely distributed across the country. In the Villeta municipality, leptospirosis has been identified as an important cause of febrile illness; however, to date, no studies have been performed to identify the circulating species. A genus-specific qualitative qPCR was performed on DNA extracted from febrile patients’ acute-phase whole-blood samples targeting a fragment of the *rrs* gene. Positive qPCR samples were further amplified for the *adk*, *icdA*, *LipL32*, *LipL41*, *rrs*, and *secY* genes through conventional PCR for sequencing. All high-quality obtained sequences were further assessed through concatenated phylogenetic analysis. A total of 25% (14/56) of febrile patients’ acute blood samples were positive for *Leptospira* spp. High-quality sequences were obtained for only five genes, and analysis through concatenated phylogeny identified that all sequences clustered within the P1/pathogenic clade; some of them formed a robustly supported clade with *Leptospira santarosai*, and others were closely related with other *Leptospira* species but exhibited considerable genetic divergence. We describe the presence of pathogenic *Leptospira* species among febrile patients from the Villeta municipality and identify *L. santarosai* and other *Leptospira* species as causative agents of leptospirosis in the region.

## 1. Introduction

*Leptospira* spp. (order: *Spirochaetales*, family: *Leptospiraceae*) is a bacterial genus that comprises long, thin, and flexible Gram-negative spirochetes, some of which are pathogenic to a great number of animal species, including humans [1,2]. More than 66 *Leptospira* species have been officially recognized, of which at least 25 are well-known pathogens related to human and animal diseases [3]. Pathogenic *Leptospira* species are maintained in nature among a wide range of animal species such as rodents, canines, ruminants, and chiropterans. These animals develop persistent kidney colonization and shed the bacteria through the urine, acting as reservoirs [4,5,6]. Traditionally classified into pathogenic and saprophytic species [7], advances in molecular and phylogenetic analysis have rearranged *Leptospira* spp. into pathogenic, intermediate, and saprophytic groups [8]. Recently, the genus has been arranged into P1, P2, S1, and S2 clades to avoid a virulence assumption of novel *Leptospira* species [3]. Both classifications are currently being used with *Leptospira* spp. known to be pathogenic clustered within the P1/pathogenic clade [3,8].

Pathogenic *Leptospira* spp. cause leptospirosis, a neglected zoonotic infectious disease widely distributed across the world and one of the main public health problems in tropical and subtropical regions, mainly in developing and underdeveloped countries [1,9,10]. Leptospirosis is one of the etiologies of acute undifferentiated febrile illness (AUFI), and in some regions, it represents even a higher concern in comparison with other outstanding febrile illnesses such as malaria and dengue as a result of its high burden of disease [11]. The global average incidence of human leptospirosis is approximately 1.9 cases per 100,000 individuals. Its prevalence ranges between 11% and 30%, being more prevalent in tropical and subtropical regions of developing and underdeveloped countries [12]. It usually affects rural inhabitants whose economic activities are related to livestock production and agriculture [13,14]. Other practices such as eco-tourism, along with environmental modifications such as accelerated urbanization and climate change, have favored greater contact with wildlife and the re-emergence of leptospirosis [15]. Leptospirosis is usually a mild and self-limiting disease characterized by a sudden onset of fever accompanied by non-specific symptomatology (e.g., headaches, muscle pain, chills). However, some patients can develop severe and fulminant forms of the disease, which are life threatening if the disease is not promptly recognized and treated. Severe cases may result in renal and liver failure, usually known as Weil’s disease, or multiple-organ damage associated with sepsis [16,17].

At least 85% of the Colombian national territory is composed of tropical and subtropical ecosystems, which highlights the relevance of tropical diseases for the country’s population and visiting foreigners [18,19]. Since 2007, leptospirosis has been considered a mandatorily reported disease for the Colombian National Public Health Surveillance System (SIVIGILA), and novel cases are continuously being reported from several regions of the country [20]. In Colombia, few studies have been performed to, among other contributions, clarify the epidemiology of the disease, establish previous exposure to *Leptospira* spp., try to identify potential animal reservoirs, or determine the circulating *Leptospira* serovars and species [21,22,23,24]. In the department of Cundinamarca (Colombian Andean region), only two studies have been performed in the municipality of Villeta, where leptospirosis was identified as the first and second cause of AUFI in 2017 and 2023, respectively. Some of the circulating serovars have been identified as causes of human illness [25,26]. However, besides these two studies, no additional information is available despite the great relevance of leptospirosis as a frequent and significant cause of AUFI in the region. Thus, to contribute to the local knowledge and data on the circulating pathogenic *Leptospira* species, we conducted a molecular characterization of *Leptospira* strains among febrile patients from the Villeta municipality.

## 2. Materials and Methods

### 2.1. Ethical Consideration

The protocol and informed consent were approved by the institutional review board (IRB) of the “Pontificia Universidad Javeriana”. Each of the recruited patients provided written informed consent voluntarily. For patients younger than 6 years old and those in critical condition, written informed consent was provided by the parents or the legal guardians. For patients older than 6 years old but younger than 18 years old, a minor assent was also obtained before parents or legal guardians signed the written informed consent form. All the information provided was treated anonymously using numerical codes for each recruited patient. All the management, procedures, and conservation of biological samples obtained from febrile patients were carried out following the norms established in the resolution No. 8430 of 1993 of the Colombian Ministry of Health and the declaration of Helsinki for ethical and medical research in human subjects.

### 2.2. Study Area

The study was performed in the municipality of Villeta (5°00′46″ N, 74°28′23″ W), located in the Gualivá province, department of Cundinamarca. The Villeta municipality comprises a total area of 140 km^2^, which is distributed across 22 villages. Geographically, it is located 84 km from Bogotá D.C. and at 850 m above sea level. Villeta has an annual mean temperature of 26 °C and a relative humidity ranging from 80% to 97%, and its economic activity depends mainly on agriculture, principally sugarcane crop and “panela” production, which is a Colombian typical natural sweetener obtained from the evaporation, concentration, and crystallization of undistilled sugar cane juice, and more recently eco-tourism for local populations as well as for international travelers (https://www.villeta-cundinamarca.gov.co/Paginas/default.aspx accessed on 31 May 2024). According to the 2018 national population and housing census of the “Departamento Administrativo Nacional de Estadística (DANE)”, Villeta has a total population of 25,957 inhabitants, of which 17,751 live in the urban area, 743 live in suburban settlements, and 7463 live in rural areas (https://www.dane.gov.co accessed on 31 May 2024).

### 2.3. Febrile Patient Recruitment

Between September and December 2021, an active surveillance of febrile patients was conducted at “Salazar de Villeta” hospital, located in the main urban area of the Villeta municipality [26]. Male and female patients older than 2 years old who presented to the emergency department due to a non-specific febrile illness of less than fourteen days of evolution without an evident source of infection were included in the study. Each patient was recruited after voluntarily accepting participation in the study and having signed a informed consent form by themselves or through parents or legal tutors when appropriate. Whole-blood samples were collected upon admission from each recruited patient (acute-phase sample) and stored at −20 °C at the “Laboratorio de Bacteriología Especial” of the Faculty of Sciences of the “Pontificia Universidad Javeriana”, Bogotá D.C., Colombia, until further processing.

### 2.4. DNA Extraction

DNA was extracted from 100 µL of febrile patients’ acute-phase whole-blood samples using the DNeasy^®^ Blood & Tissue Kit (Qiagen^®^, Hilden, Germany) following the manufacturer’s instructions. After each extraction procedure, DNA purity, to rule out the presence of inhibitors, and quantity were evaluated using a NanoDrop 2000 instrument (Thermo Scientific, Wilmington, DE, USA). DNA integrity was evaluated by performing a conventional PCR targeting a 289 base pair (bp) fragment of the β-actin (*ACTB*) gene using the primers Actin-FWD (CGGAACCGCTCATTGCC) and Actin-REV (GCTCACTCAGTGTGGCAAAG) according to previously reported procedures [27]. The PCR protocol was performed through 40 amplification cycles in a 96-well T100 PCR thermal cycler using the GoTaq^®^ Green Master Mix (Promega Corporation, WI, USA) and employing the volumes and concentrations of Master Mix, primers, and DNA suggested by the Master Mix manufacturer’s instructions for a 10 μL reaction volume. Positive (genomic human DNA) and negative (molecular-grade water) controls were included in all amplification procedures. All amplified products were subsequently visualized on a 1% agarose gel stained with SYBR^TM^ Safe DNA Gel Stain (Invitrogen, Waltham, MA, USA). Samples positive for the *ACTB* gene were further screened for the presence of *Leptospira* spp.

### 2.5. Detection of Leptospira spp.

For detecting the presence of *Leptospira* spp. DNA, a genus-specific qualitative real-time PCR (qPCR) was performed on DNA extracted from the blood samples of febrile patients during the acute phase. The qPCR test targeted a 331 bp fragment of the 16S rRNA- encoding gene (*rrs*) using the primers Lep1 (GGCGGCGCGTCTTAAACATG) and Lep2 (TTCCCCCCATTGAGCAAGATT) [28]. The PowerUp^TM^ SYBR^TM^ Green Master Mix (Applied Biosystems, Austin, TX, USA) was used to set up all reactions.

qPCR samples positive for the leptospiral *rrs* gene were further amplified using a conventional PCR method with the same set of primers for sequencing procedures. The PCR protocol was also performed through 40 amplification cycles in a 96-well T100 PCR thermal cycler using the GoTaq^®^ Green Master Mix and following the recommendations for a 10 μL reaction volume. Amplicons were also evaluated in a 1% agarose gel run by electrophoresis and stained with SYBR^TM^ Safe DNA Gel Stain. For both procedures, a positive control, *Leptospira interrogans* serovar Icterohaemmorhagiae DNA, and a negative control, molecular-grade water, were employed for all amplification reactions. All obtained amplicons from the *Leptospira rrs* protocol were purified using a Wizard^®^ DNA Clean-Up System Kit (Promega, Madison, WI, USA), and then bi-directionally sequenced employing a 3500 Genetic Analyzer (Applied Biosystems, Foster City, CA, USA).

### 2.6. Identification of Leptospira spp.

To identify the *Leptospira* strains detected from febrile patient samples, a multi-locus sequence typing (MLST) approach was performed through conventional PCR on all DNA from febrile patients’ acute-phase blood samples that were positive for the leptospiral *rrs* gene, targeting five different genes: a 531 bp fragment of the adenylate kinase-encoding gene (*adk*), a 674 bp fragment of the isocitrate dehydrogenase-encoding gene (*icdA*), a 474 bp fragment of the major outer membrane lipoprotein lipL32-encoding gene (*LipL32*), a 520 bp fragment of the outer membrane lipoprotein lipL41-encoding gene (*LipL41*), and a 549 bp fragment of the pre-protein translocase-encoding gene (*secY*), using the sets of primers reported elsewhere [29]. The same positive and negative controls used in the *Leptospira rrs* screening protocol were also used for all five complementary gene amplification procedures. The PCR protocol was also performed through 40 amplification cycles in a 96-well T100 PCR thermal cycler using the GoTaq^®^ Green Master Mix and following the recommendations for a 10 μL reaction volume. PCR products were also evaluated by electrophoresis in a 1% agarose gel stained with SYBR^TM^ Safe DNA and then purified and bi-directionally sequenced, similarly to the amplicons obtained from the *Leptospira rrs* screening protocol. All six sequenced amplified molecular markers (*adk*, *icdA*, *LipL32*, *LipL41*, *rrs*, and *secY*) were used for further concatenated phylogenetic analyses.

### 2.7. Phylogenetic Analyses

The forward and reverse sequences were assembled and/or edited using SnapGene^®^ Viewer 6.0.5 software and then compared with the NCBI GenBank sequence database using the BLASTn server. *Leptospira* reference sequences were retrieved from all *Leptospira* species available in the NCBI nucleotide sequence repository for all the six molecular markers (*adk*, *icdA*, *LipL32*, *LipL41*, *rrs*, and *secY*) analyzed in the present study.

For each individual marker, the successfully sequenced *Leptospira*-positive samples and reference sequences were aligned with the ClustalW algorithm [30], and then the alignment matrices were concatenated using the MEGA X software version 10.0.5 [31]. A maximum-likelihood (ML) tree was built with the consensus matrix in Randomized Axelerated Maximum Likelihood (RAxML) [32] using the GTR+GAMMA evolutionary model, which was identified as the best model according to the Bayesian information criterion, and a branch-support analysis of 1000 bootstrap replicates.

## 3. Results

### 3.1. Detection of Leptospira spp. in Febrile Patients

DNA extraction was performed on 56 febrile patients’ acute blood samples. The *ACTB* gene was successfully amplified in all processed samples, as expected. A total of 25% (14/56) of febrile patients’ acute blood samples were positive based on the qPCR screening targeting the leptospiral *rrs* gene. All amplified samples had Ct values ranging from 27.36 to 32.67 and melting temperatures (Tm) oscillating between 77.84 and 85.62. An expected amplicon of 331 bp was obtained in all 14 positive samples through conventional PCR targeting the same gene.

All 14 qPCR-amplified samples were tested for the five selected complementary genes, of which 9 were amplified for the leptospiral *secY* gene, 5 for the leptospiral *LipL32* gene, 4 each for the leptospiral *adk* and *icdA* genes, and only 1 for the leptospiral *LipL41* gene.

The detection of *Leptospira* spp. was more frequent in male patients [33.3% (10/30)] and among children 3-12 years of age [83.3% (5/6)]. Most *Leptospira* spp.-positive patients lived in rural areas [44.4% (4/9)] and were recruited during October [71.4% (5/7)] and September [66.7% (4/6)]. Detailed information is provided in Table 1. In five of the fourteen positive febrile patients, positive serological results were obtained previously [26] through the detection of IgM antibodies in acute and/or convalescent serum samples; and in one of them, seroconversion to serovars Brastislava and Hardjo was evidenced (Table 2).

### 3.2. Molecular Identification of Leptospira spp. in Febrile Patients

In order to identify the species involved in human leptospirosis in the municipality of Villeta, a maximum-likelihood phylogeny of *Leptospira* spp. was generated using an MLST profile. All obtained *Leptospira rrs* amplicons were sequenced, and 13/14 had high enough quality to be further analyzed through phylogenetic analysis. Regarding the other five selected complementary genes, all obtained amplicons were also sequenced, but high-quality sequences were only obtained for four of them: *icdA* (2/4), *LipL32* (3/5), *LipL41* (1/1), and *secY* (4/9); thus, reference sequences of the *Leptospira adk* gene were excluded from further analyses. High-quality obtained sequences belonged to four different patients’ samples as follows: COV009 (*icdA* and *secY*), COV013 (*secY*, *LipL32*, and *LipL41*), COV021 (*icdA*, *secY*, and *LipL32*), and COV023 (*secY* and *LipL32*). All of them were further analyzed through phylogeny using the obtained *Leptospira* spp. reference sequences for the *icdA*, *LipL32*, *LipL41*, and *secY* genes (Table S1).

**Figure 1:**
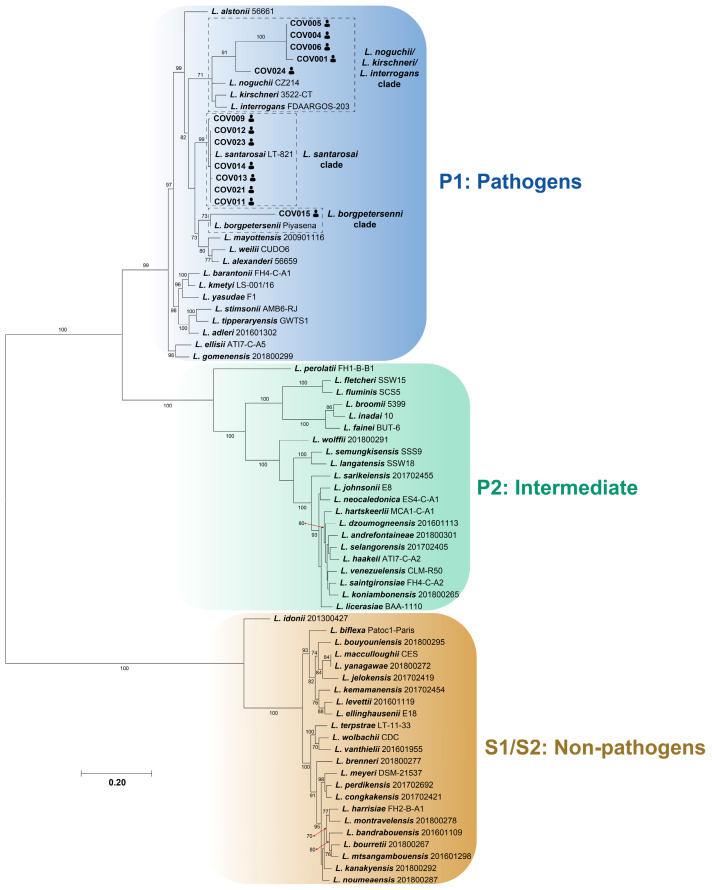
Maximum-likelihood phylogeny of *Leptospira* spp. based on concatenated sequences (*rrs*, *icdA*, *secY*, *LipL32*, or *LipL41*) detected in patients with leptospirosis from Villeta municipality. Sequences are signaled by human icons, and *Leptospira* groups are indicated by colored boxes as follows: blue—P1 or pathogenic *Leptospira* spp.; green—P2 or intermediate *Leptospira* spp.; orange—S1/S2 or non-pathogenic *Leptospira* spp. Clades with *Leptospira* sequences obtained from febrile patients from the municipality of Villeta mixed with or related to *Leptospira* species reference sequences are denoted by dashed boxes. Only bootstraps higher than 70% are shown. A more extensive description of the sequences used for each human sample or *Leptospira* reference genome can be found in Table S1.

All acquired sequences were clustered within the *Leptospira* P1/pathogenic clade (Figure 1). The analyzed sequences were closely related to specific *Leptospira* species: seven sequences (COV009, COV011, COV012, COV013, COV014, COV021, COV023) grouped with *Leptospira santarosai*, including those with more than one successfully identified sequence molecular marker (COV009, COV013, COV021, COV023), forming a robustly supported clade and indicating a close relationship with the reference species due to the short branch distances (Figure 1). Five sequences (COV001, COV004, COV005, COV006, and COV024) clustered together, forming a clade closely related to *Leptospira noguchii*, *Leptospira kirschneri*, and *Leptospira interrogans* but exhibiting considerable genetic divergence from these three reference species (Figure 1). Finally, an additional sequence (COV015) formed a clade with *Leptospira borgpetersenii*, also showing a notably high branch distance (Figure 1).

Furthermore, a BLAST analysis was also performed on all good-quality sequences obtained in the present study. The BLAST analysis of *Leptospira rrs* sequences showed that all of them had a high similarity to sequences of recognized human-pathogenic *Leptospira* species (*L. borgpetersenii*, *L. interrogans*, *L. kirschneri*, *L. noguchii*, and *L. santarosai*). In some cases, a sequence obtained from febrile patients presented high similarity with sequences from more than one *Leptospira* species. The BLAST analysis of all *Leptospira icdA*, *LipL32*, *LipL41*, and *secY* sequences showed a high percentage of identity only with sequences of *L. santarosai*. More information can be observed in Table 2.

## 4. Discussion

The municipality of Villeta has been recognized for several years as an endemic region for spotted fever group rickettsiosis, where *Rickettsia rickettsii* is actively circulating [33,34]. However, two studies performed in the region that aimed to characterize the etiology of AUFI have identified other diseases such as dengue and leptospirosis as important causes of febrile illnesses [25,26]. Despite the importance of spotted fever group rickettsiosis, in one of these studies, leptospirosis was identified as the main and most frequent cause of AUFI [25], which highlights the importance of this zoonotic disease in the region. However, despite the surveillance performed by the Colombian national surveillance system, nothing is known regarding the circulating *Leptospira* strains associated with human leptospirosis in the region. The present study fills this gap by assessing the presence of pathogenic *Leptospira* species among febrile patients recruited in the municipality of Villeta and identifying them as *L. santarosai* and other species closely related to recognized human-pathogenic *Leptospira* species as causes of leptospirosis in the region.

Throughout the world, in several tropical and subtropical regions, leptospirosis has been recognized as one of the most important and frequent causes of febrile illness, as well as the most widespread zoonosis worldwide. It has also gained great relevance due to its re-emergence in several regions of different countries, becoming a major public health problem [10,35]. Since 2007, leptospirosis has been a mandatorily reported disease for the Colombian National Surveillance System (SIVIGILA) due to the unusual increase in reported cases throughout the national territory [36]. Cases of leptospirosis have been reported in all 32 departments of Colombia since 2007 until 2023; however, the largest number of cases was concentrated and reported in the department of Antioquia (https://www.ins.gov.co accessed on 30 May 2024), including municipalities from the Urabá gulf region, which is the most important and main endemic region for leptospirosis in Colombia [37]. Regarding the department of Cundinamarca, a total of 8.5% of the reported cases of leptospirosis by the national surveillance system between 2015 and 2020 came from this region [20]. In the year 2023, a total of 202 suspected cases were reported in the department of Cundinamarca, representing the 2.9% of cases reported throughout the country. For the first four epidemiological weeks of 2024, 65 suspected cases were already reported, which represent the 2.7% of all reported cases in the country, suggesting an increase in suspected cases of leptospirosis in the department of Cundinamarca (http://www.ins.gov.co/buscador-eventos/Informesdeevento accessed on 30 May 2024).

Despite the available data from the national surveillance system, few studies have been performed in regions in which leptospirosis has been identified as an important cause of AUFI. In the department of Valle Del Cauca, leptospirosis was confirmed in 20.6% (31/150) of suspected cases reported between 2003 and 2006 by different healthcare centers of the public network of the department [38]. In the Urabá region of the department of Antioquia, leptospirosis was identified as the cause of AUFI in 14.1% (31/220) of febrile patients recruited from three different municipalities [39]. In the Córdoba department, leptospirosis was identified as the etiology in 39.1% (27/69) of recruited AUFI patients from a main hospital of the region [40], and more recently, acute leptospirosis was confirmed in 19.8% (67/339) of suspected cases from two health service provider institutions of the department [24].

In the municipality of Villeta, Cundinamarca, leptospirosis was identified as the cause of AUFI in 24% (25/104) and 21.4% (12/56) of recruited patients in 2017 and 2023, respectively [25,26]. In the last study, only five of the twelve identified cases were detected through conventional PCR. Considering the recommendations published elsewhere [41], and considering that the use of qPCR is a more sensitive method than its conventional variant [42], in the present study, we decided to use a qualitative qPCR screening method on the same samples to reduce the underreporting of cases. We detected the presence of *Leptospira* spp. in fourteen febrile patients’ acute blood samples, of which five reported cases were already diagnosed through conventional PCR and one reported case through ELISA IgM and confirmed with seroconversion [26], yielding a total of eight more cases that were initially unreported.

Furthermore, through sequencing analyses performed on these samples, thirteen high-quality sequences were obtained for the *Leptospira rrs* gene, which were greatly similar to *L. borgpetersenii*, *L. noguchii*, *L. kirschneri*, and *L. santarosai*. Furthermore, in a second step using an MLST approach targeting five complementary genes, good-quality sequences were obtained from four processed samples, and through phylogenetic analyses, *L. santarosai* was identified as the infectious species in these samples. Previous studies have shown that by far the most studied species in terms of virulence, epidemiology, and other characteristics was *L. interrogans*, probably due to its widespread distribution, leaving behind other *Leptospira* species that are more geographically restricted, for which little is known to date [43]. One of these species is *L. santarosai*, described in 1987 and named in honor of Carlos A. Santa Rosa, a Brazilian veterinary microbiologist and pioneer in the study of leptospirosis in that country, and is a recognized pathogenic species associated with both human and animal leptospirosis [44,45,46]. Although *L. santarosai* has been recognized as an important agent of leptospirosis in several countries in Central and South America [47], its presence has also been reported in a few regions outside the American continent, such as Sri Lanka, India, and Taiwan [48,49,50]. This suggests that this *Leptospira* sp. is more widespread than what is known to date. Available information has revealed that *L. santarosai* has been isolated from a few domestic and wild animal species such as dogs and capybaras [51,52]. However, ruminant species such as bovines and goats appear to be the most important hosts of this *Leptospira* sp. [53,54,55], representing a high risk of exposure for populations that work with cattle. In Colombia, this species has already been described as an agent of human and canine leptospirosis [56,57].

The remaining nine additional positive *Leptospira rrs* samples in which a sequence was obtained (COV001, COV004, COV005, COV006, COV011, COV012, COV014, COV015, and COV024) could not be amplified for any complementary gene; however, through phylogenetic analyses, three of them also showed a strong relationship with *L. santarosai* and probably belong to that species. Of the remaining six sequences, five formed a clade within *L. interrogans*, *L. kirschneri*, and *L. noguchii* species, all of which have already been recognized as pathogenic species associated with human and animal leptospirosis [7,58,59,60]. One last sequence formed a clade related with *L. borgpetersenii*, a well-known pathogenic species associated with the intensification of agriculture patches and animal farms [6,61,62]. However, considering that these isolated sequences exhibited considerable genetic divergence and a notably high branch distance from reference sequences, in addition to only being amplified for a single targeted gene, it was not possible to identify the specific *Leptospira* species. It is probable that these sequences belong to novel, unrecognized species highly related to human-pathogenic *Leptospira* species. Atypical genomic features have been identified among a few human-pathogenic *Leptospira* species, which suggests an ongoing evolution of this group of leptospires and a diversification of lineages that also impacts in their pathogenicity. Therefore, specific genetic determinants that have undergone positive selection during *Leptospira* evolution not only favor their diversification into novel species and strains but also contribute directly to their virulence [3,63]. Thus, understanding the eco-epidemiology of leptospirosis and its transmission dynamics [64] in endemic areas is a crucial step in creating prevention and control measures.

## 5. Conclusions

The present study described the presence of pathogenic *Leptospira* spp. among febrile patients from the Villeta municipality of Cundinamarca, Colombia. It also identified *L. santarosai* as one of the causative agents of leptospirosis in the region, as well as two different *Leptospira* strains closely related to recognized human-pathogenic *Leptospira* species, which are probably novel species. These data reinforce the importance of leptospirosis as one of the common causes of AUFI, highlight the importance of *L. santarosai* as perhaps the main *Leptospira* species associated with human leptospirosis in this region, and provide preliminary evidence of other *Leptospira* species associated with human disease that are not yet officially recognized. Further studies are needed in the region to better understand the epidemiology of leptospirosis associated with *L. santarosai* and other *Leptospira* species in the region and create appropriate preventive and control measurements to avoid infection.

## Figures and Tables

**Table 1:** Detection of *Leptospira* spp. considering the demographic characteristics of recruited febrile patients in the municipality of Villeta.

Demographic Characteristic	n (%)	*Leptospira* spp. Detection (%)
**No. of patients**	56 (100)	14 (25)
**Gender**		
Male	30 (53.6)	10 (33.3)
Female	26 (46.4)	4 (15.3)
**Age groups**		
Children (3–12)	6 (10.7)	5 (83.3)
Adolescents (13–16)	4 (7.1)	1 (25)
Young adults (17–29)	21 (37.5)	4 (19)
Middle-aged adults (30–44)	12 (21.5)	2 (16.7)
Older adults (above 45)	13 (23.2)	2 (15.4)
**Origin**		
Urban area	47 (83.9)	10 (21.3)
Rural area	9 (16.1)	4 (44.4)
**Sampling month**		
September	6 (10.7)	4 (66.7)
October	7 (12.5)	5 (71.4)
November	10 (17.9)	4 (40)
December	33 (58.9)	1 (3)

**Table 2:** Description of the BLASTn results using *Leptospira rrs*, *secY*, *LipL32*, *LipL41*, and *icdA* sequences obtained from febrile patients’ samples and their relations with previously reported serological results.

Sample ID	Gene	BLASTn Results					Serological Results from Silva-Ramos et al. 2023 [26]
		Organism	Identity (%)	Coverage (%)	e-Value	GenBank ID	
COV001	*rrs*	*L. noguchii*	96.3%	100.0%	1 × 10^−91^	CP091936.1	Positive IgM in acute and convalescent samples Seroconversion to serovar Bratislava Seroconversion to serovar Hardjo
COV004	*rrs*	*L. noguchii*	96.7%	100.0%	4 × 10^−91^	CP091936.1	
COV005	*rrs*	*L. noguchii*	96.4%	100.0%	4 × 10^−96^	CP091936.1	
COV006	*rrs*	*L. noguchii*	96.4%	100.0%	3 × 10^−82^	CP091967.1	
COV009	*rrs*	*L. santarosai*	100.0%	100.0%	2 × 10^−88^	MH801931.1	Positive IgM in convalescent sample
		L. interrogans *	100.0%	100.0%	2 × 10^−88^	MH686123.1	
	*secY*	*L. santarosai*	100.0%	100.0%	6 × 10^−169^	MK315145.1	
	*icdA*	*L. santarosai*	99.7%	99.0%	4 × 10^−175^	KC492816.1	
COV011	*rrs*	*L. santarosai*	100.0%	100.0%	9 × 10^−103^	MH801931.1	
		L. interrogans *	100.0%	100.0%	9 × 10^−103^	MH686123.1	
COV012	*rrs*	*L. santarosai*	100.0%	100.0%	9 × 10^−103^	MH801931.1	
		L. interrogans *	100.0%	100.0%	9 × 10^−103^	MH686123.1	
COV013	*rrs*	*L. santarosai*	100.0%	100.0%	9 × 10^−103^	MH801931.1	Positive IgM in convalescent sample
		L. interrogans *	100.0%	100.0%	9 × 10^−103^	MH686123.1	
	*secY*	*L. santarosai*	99.7%	100.0%	0	EU358050.1	
	*LipL32*	*L. santarosai*	100.0%	100.0%	9 × 10^−116^	PP554251.1	
	*LipL41*	*L. santarosai*	99.5%	100.0%	0	AY461959.1	
COV014	*rrs*	*L. santarosai*	100.0%	100.0%	5 × 10^−90^	MH801931.1	Positive IgM in convalescent sample
		L. interrogans *	100.0%	100.0%	5 × 10^−90^	MH686123.1	
COV015	*rrs*	*L. borgpetersenii*	94.7%	100.0%	3 × 10^−66^	CP047520.1	
COV021	*rrs*	*L. santarosai*	100.0%	100.0%	9 × 10^−103^	MH801931.1	
		L. interrogans *	100.0%	100.0%	9 × 10^−103^	MH686123.1	
	*secY*	*L. santarosai*	100.0%	100.0%	6 × 10^−148^	MK315143.1	
	*LipL32*	*L. santarosai*	100.0%	100.0%	7 × 10^−117^	AY461928.1	
	*icdA*	*L. santarosai*	100.0%	100.0%	0	CP028377.1	
COV023	*rrs*	*L. santarosai*	100.0%	100.0%	1 × 10^−96^	MH801931.1	Positive IgM in convalescent sample
		L. interrogans *	100.0%	100.0%	1 × 10^−96^	MH686123.1	
	*secY*	*L. santarosai*	100.0%	100.0%	0	MK315138.1	
	*LipL32*	*L. santarosai*	100.0%	100.0%	7 × 10^−153^	PP554251.1	
COV024	*rrs*	*L. kirschneri*	98.1%	99.0%	1 × 10^−96^	CP125672.1	
		*L. interrogans*	98.1%	99.0%	1 × 10^−96^	KP211707.1	

* MH686123.1 was the only *L. interrogans* sequence in the NCBI nucleotide database that matched through BLASTn analysis with the selected sequences listed in the present study. MH686123.1 is not linked to any published manuscript that allows for validation of the methodology used.

## Data Availability

The data generated and analyzed during the current study are not publicly available, but they can be shared upon request to the corresponding authors.

## Supplementary Materials

The following supporting information can be downloaded at: https://www.mdpi.com/article/10.3390/tropicalmed9080168/s1: Table S1: Database used for the concatenated phylogenetic analysis performed on *Leptospira* obtained sequences from patients with febrile patients from the Villeta municipality, Cundinamarca department, Colombia.
